# Antibiotic Resistance and Biofilm Production in *Staphylococcus epidermidis* Strains, Isolated from a Tertiary Care Hospital in Mexico City

**DOI:** 10.1155/2013/918921

**Published:** 2013-02-03

**Authors:** Roberto Cabrera-Contreras, Rubén Morelos-Ramírez, Ada Nelly Galicia-Camacho, Enrique Meléndez-Herrada

**Affiliations:** ^1^Department of Public Health, Faculty of Medicine, National Autonomous University of Mexico, 04510 Mexico City, DF, Mexico; ^2^Department of Microbiology and Parasitology, Faculty of Medicine, National Autonomous University of Mexico, 04510 Mexico City, DF, Mexico

## Abstract

*Staphylococcus epidermidis* strains isolated from nosocomial infections represent a serious problem worldwide. In various Mexican states several reports have shown isolates from hospitals with antibiotic resistance to methicillin. In Mexico City, there is scarce information on staphylococcal infections in hospitals. Here, our research findings are shown in a four-year period study (2006–2010) for *Staphylococcus epidermidis* strains. Susceptibility and/or resistance to antibiotics in SE strains were assessed by phenotypic and molecular methods as *mecA* gene by PCR, as well as the correlation with biofilm production for these isolates and the relationship to the infection site. Out of a total of 161 (66%) negative biofilm SE strains, just 103 (64%) SE strains were confirmed as MRSE by PCR to *mecA* gene. From 84 (34%) positive biofilm SE strains, 76 (91%) were confirmed as MRSE by PCR to *mecA* gene. Higher percentages of resistance to antibiotics and higher number of resistance markers were found in biofilm-forming clinical strains (9 to 14) than non-biofilm-forming SE strains (3 to 8). These research findings represent a guide to establish infection control programs for this hospital.

## 1. Introduction

*Staphylococcus epidermidis* (SE) is a saprophyte which is part of the normal human skin and mucous membranes microflora. Furthermore, SE is one of the most common etiologic agents of nosocomial infections (NIs) worldwide. Moreover, SE is the most prevalent etiology of NI in pediatric intensive care units (ICUs) in Mexican hospitals [1, 2].

However, SE is primarily associated with infections in patients implanted with medical devices, such as prosthetic heart valves and orthopedic prostheses, mainly in immunocompromised children. Furthermore, SE was isolated with higher incidence to catheter-related bacteremia in a report from secondary care Hospital in Durango, México [3, 4]. 

Biofilm is mainly made of polysaccharide component; it seems to be the most important factor by which SE adheres to and colonizes artificial materials (catheters) commonly implanted in patients with NI. Biofilm is believed to make clinical SE strains more resistant to administered antibiotics and to host defense mechanisms and highly contributed to cause NI in patients [2, 5–9]. 

In clinical practice, SE has become one of the most significant species among methicillin-resistant coagulase negative staphylococci (CoNS). There are various scientific world reports that stated that approximately between 80% and 90% of SE strains isolated from patients with NI carried the *mecA* gene [10–13]. The presence of *mecA* gene seems to be enhanced in biofilm producers and enable SE strains to show increasing resistance to different groups of antibiotics [14, 15].

In a university hospital at Monterrey, Mexico, staphylococci methicillin-resistant strains were detected by molecular methods, and SE was the most common species identified [16]. A general hospital of Mexico City performs a series of molecular tests to determine *icaA* gene and consequently biofilm production from staphylococci isolates and research findings here show that SE biofilm producers were the most common species found [17].

Actually, there are only few hospital reports that express the genuine situation of SE infections in Mexico, which means that more research is required to understand the real scenario about NI in other hospitals, especially in Mexico City, which is one of the biggest and crowdest cities in the world. The aim of the present study is the phenotypic and genotypic characterization related to biofilm production, methicillin and other antibiotic resistance patterns to SE strains isolated from NI in a tertiary-care-level hospital in Mexico City.

## 2. Methods

A total of 245 strains were isolated from patients with NI from a tertiary-care-level hospital during a four-year period (2006–2010). The biotype and antibiotype for seventeen antimicrobial agents were performed by an automated Micro Scan System (American Micro Scan, Mahwah, NJ), using a specific panel set (Positive BP Combo 20; DADE Behring, Sacramento, CA.) as follows: Amoxicillin/Clavulanic acid “AMC,” Ampicillin “AMP,” Cefazolin “KZ,” Cefotaxime “CTX”, Ciprofloxacin “CIP”, Clindamycin “DA,” Chloramphenicol “C,” Erythromycin “E,” Gentamicin “CN,” Imipenem “IPM,” Levofloxacin “LEV,” Oxacillin “OX,” Penicillin “P,” Rifampin “RD,” Tetracycline “TE,” Trimethoprim/Sulphamethoxazole “SXT,” and Vamcomycin “VA.” These antibiotics above represent common antibiotics used in Mexican Hospitals. All strains in this study were stored frozen in 20% skim milk at −80°C until use. 

### 2.1. *mecA* PCR Assay

PCR assay was performed for all 245 SE strains searching an amplification product of 458 bp that represent a fragment of *mecA* gene. The PCR primers used were 5′-ATGGCAAAGATATTCAACTA-3′ (upstream) and 5′-GAGTGCTACTACTCTAGCAAAGA-3′ (downstream). The primers were designed for *mecA* gene by our group using a Primer3 free software (http://frodo.wi.mit.edu/primer3/) and the information available in GenBank/NCBI (http://www.ncbi.nlm.nih.gov/) to genome sequence for SE strain RP62A (accession NC_002976) the primer sequences for* mecA* gene were compared with other staphylococci genomes using the BLAST algorithm (http://blast.ncbi.nlm.nih.gov/Blast.cgi) then our PCR primers were considered specific to this gene.

 Ten to twenty colonies of each SE isolate grown on BHI agar plates were suspended in 250 *μ*L of lysis buffer solution (0.01 M Tris-HCL SIGMA-ALDRICH, Co. St. Louis MO; 0.01 M EDTA SIGMA-ALDRICH, Co. St. Louis MO; lysozyme (200 *μ*g/mL; SIGMA-ALDRICH, Co., St. Louis MO) and lysostaphine (20 *μ*g/mL; Sigma-Aldrich, Co., St. Louis MO)); this bacterial suspension was incubated at 37°C during 90 minutes then after the samples were warmed up at 95°C for 10 minutes and finally were centrifuged at 8000 ×g (Spectrafuge 16 M, Labnet International, Inc., Edison, NJ, USA). The supernatant was recovered in aseptic conditions, and DNA was semipurified after precipitation with isopropanol at −20°C (HPLC 99.5%, Tecsiquim, SA de CV, México, DF).

The PCR was performed in an MJ Research PT-200 equipment (GMI, Inc., Ramsey, MN, USA). The experimental conditions used were as follows: an initial denaturation phase at 94°C for 10 minutes, then 30 cycles of 94°C for 30 seconds, annealing at 49.7°C for 30 seconds, and extension at 72°C for 30 seconds, with a final primer extension at 72°C for 10 min. The PCR products were separated by electrophoresis through 1% agarose gels in 1X TAE buffer (40 mM Tris Acetate, 1 mM EDTA; Invitrogen Life Technologies Carlsbad, CA, USA) run the gels for 40 minutes at 95 Volts. The gels were stained with 0.08 *μ*L/mL of ethidium bromide (10 mg/mL; Invitrogen Life Technologies Carlsbad, CA, USA), and bands were visualized under UV light.

### 2.2. Biofilm Test

The biofilm production for SE clinical isolates was assessed by Congo Red Agar plates assay (CRA). This culture medium (CRA) was prepared with Brain Heart Infusion agar (BHI) (OXOID LTD., Basingstoke, Hampshire, England) supplemented with Congo Red dye; 0.8 g/L (Sigma-Aldrich, Co., St. Louis, MO) and sucrose; 36 g/L (J. T. Baker, México). All clinical SE strains to be tested for biofilm formation were inoculated on CRA plates. These CRA plates were incubated at 35°C for 24 hrs then after the plates were maintained at room temperature for 24 hrs. CRA plates were checked for black colonies which represented the SE biofilm producers or positive (B+), in contrast to the red colonies, which represented the SE non-biofilm producers or negative (B−) [18]. 

## 3. Results

**Figure 1:**
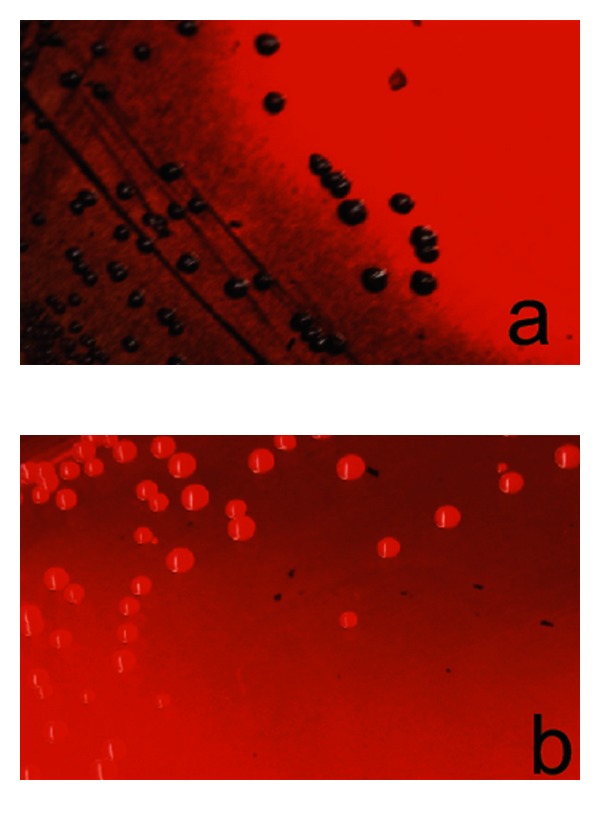
Growth of *S. epidermidis* strains on CRA plates. (a) Black colonies of SE biofilm producer or positive strains. (b) Red colonies of SE non-biofilm producer or negative strains.

From 245 SE clinical isolates, 34% (84) were detected as black colonies or biofilm-forming strains on CRA plates. The remaining SE strains 66% (161) were grown as red colonies on these plates and therefore were considered as non-biofilm producers or negative strains (Figures 1(a) and 1(b)).

Patterns of susceptibility/resistance to antibiotics of a total of 245 SE strains were performed by automated Micro Scan system. SE clinical strains of biofilm producers were displayed lightly more resistant to 14 antimicrobial agents tested than SE strains of non-biofilm producers (Table 1).

From 34% (84) positive biofilm SE strains, 91% (76) were confirmed as MRSE by PCR *mecA* gene. Out of a total of 66% (161) negative SE isolates, 64% (103) strains were confirmed as MRSE by PCR to *mecA* gene (Table 2).

According to the infection sites all the clinical SE strains were characterized by CRA plates assay. A total of 90 out of 245 (36%) SE strains were isolated from catheters 37 out of 90 (41%) were assessed as biofilm producers the majority of these 36 (97%) SE strains were *mecA* gene positive or confirmed as MRSE. A total of 71 SE isolates were recovered from bloodstream infections (BSI), 28% (20/71) were biofilm-forming strains, and 95% (19/20) were MRSE. From 27 SE strains isolated from urinary tract infections (UTIs) 33% (9/27) were biofilm formers, and from these strains 67% (6/9) were confirmed as MRSE. From the last group, including other infection sites, 57 SE strains were isolated, and 32% (18/57) were biofilm producers and 83% (15/18) of these SE strains were confirmed as MRSE (Table 2).

**Figure 2:**
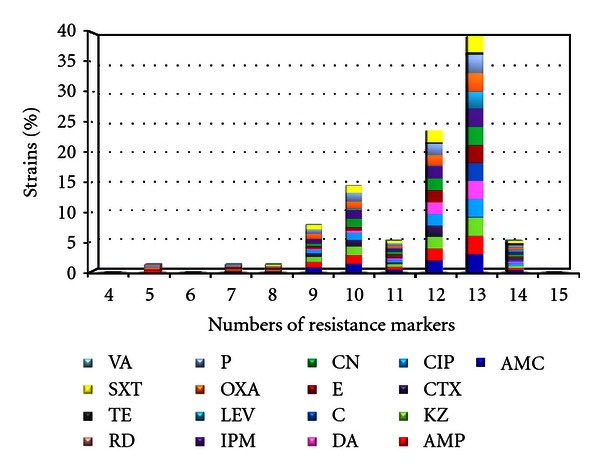
Distribution of resistance markers of *S. epidermidis *biofilm producers in *mecA* (+) strains.

**Figure 3:**
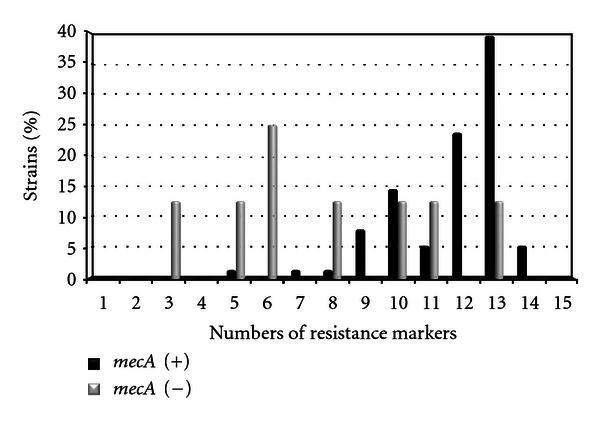
Comparison of resistance markers among *S. epidermidis* biofilm producer *mecA* (+) versus *mecA* (−) strains.

Higher percentages of resistance to antibiotics and higher number of resistance markers were found in biofilm producer *mecA* (+) clinical strains (9 to 14) than non-biofilm producer *mecA* (−) strains (3 to 8) (Figures 2 and 3).

The percentage of antibiotic resistance in SE biofilm producer strains is more relevant in the 40% of the *mecA* gene positive strains mainly in those that present resistance to 13 resistant markers. In *mecA* negative strains are appreciated for strains with 6 resistance markers corresponding to 25% of strains (Figure 3).

## 4. Discussion

In the present study we observed a quite good correlation in antibiotic resistance and biofilm production for clinical MRSE strains from patients with NI at this tertiary care hospital in Mexico City. The majority of these clinical SE strains were positive for molecular detection of the *mecA* gene by PCR. Data from other hospitals in Mexico, such as one report by Diemond et al. [17], determined the presence of biofilm in SE and CoNS strains for *icaA* operon by PCR method. In this study SE biofilm producer SE strains were found in higher percentages in catheter sites. In our study 90 out of 245 strains were isolates from catheter site, and 37 out of 90 strains were positive for biofilm production. Calderón et al. [19] in a pediatric hospital of Mexico City found resistance in 53.4% of CoNS strains to oxacillin. Other groups such as Garza et al. [16], in a tertiary care hospital, made a surveillance study for the distribution of SCCmec cassettes and its relationship to MRCoNS strains; they found that SE was the most common species identified and carrying the cassettes type III, IVa, and V for methicillin resistance. In a tertiary care hospital a study was performed by Kato-Maeda et al. [20] group, describing the resistance to antibiotics in some isolates causing bacteremia during a six-year study period (1995–2000), SE was the third most frequently isolated bacteria and the oxacillin resistance pattern was increased from 41 % to 61% during this period [8, 9].

Our research findings show that MRSE strains carry a higher number of resistance determinants in biofilm-producer strains. These strains were more frequently isolated in clinical samples from intravascular catheters in this tertiary care hospital. 

Profiles of resistance to 17 antibiotics tested were slightly higher in SE strains of biofilm producers than in non-producer strains.

The phenotypic biofilm characterization by CRA plates assay demonstrated a simple, rapid, cheap, and reliable approach for detection of biofilm producers in SE isolates from patients with staphylococci NI. It is highly recommended for the future a validation of this test *versus *genotypic test such as PCR detection for *icaA* and *icaD* genes. This molecular PCR methodology will be soon applied in this hospital for detection of other relevant antibiotic resistance markers, in order to establish a permanent NI-control program. 

The PCR *mecA *gene detection assay meets our requirements as a reliable molecular method for characterization of methicillin-resistant SE strains. Both methods are urgently needed for molecular epidemiology studies and outbreak control in Mexican hospitals. Finally our data suggest that this methodology could be used to establish infection control procedures for this tertiary care hospital in order to prevent nosocomial infections.

## Figures and Tables

**Table 1:** Resistance patterns of SE clinical strains (%) by comparison of biofilm producers versus non-biofilm producers.

Antimicrobial agents	Biofilms producers (%)	Non-biofilm producers (%)
AMC	94	78
AMP	100	97
KZ	94	78
CTX	41	29
CIP	81	46
DA	76	49
C	40	26
E	84	63
CN	87	61
IPM	93	78
LEV	42	27
OX	94	78
P	99	96
RD	1	3
TE	13	24
SXT	86	54
VA	0	0

**Table 2:** Distribution of *mecA* gen in *S. epidermidis* biofilm producer strains by diverse infection sites.

Catheter		Blood		Urine		Other	
*n* = 90		*n* = 71		*n* = 27		*n* = 57	
(90/245) = 37%		(71/245) = 29%		(27/245) = 11%		(57/245) = 23%	
Biofilm (+)	*mecA* (+)	Biofilm (+)	*mecA *(+)	BIofilm (+)	*mecA* (+)	Biofilm (+)	*mecA *(+)
*n* = 37	*n* = 36	*n* = 20	*n* = 19	*n* = 9	*n* = 6	*n* = 18	*n* = 15
(37/90) = 41%	(36/37) = 97%	(20/71) = 28%	(19/20) = 95%	(9/27) = 33%	(6/9) = 67%	(18/57) = 41%	(15/18) = 83%
